# Unhealthy lifestyle impacts on biological systems involved in stress response: hypothalamic–pituitary–adrenal axis, inflammation and autonomous nervous system

**DOI:** 10.1097/YIC.0000000000000437

**Published:** 2022-11-11

**Authors:** Laura Mandelli, Yuri Milaneschi, Sarah Hiles, Alessandro Serretti, Brenda W. Penninx

**Affiliations:** a Department of Biomedical and Neuromotor science, University of Bologna, Bologna, Italy; b Department of Psychiatry, Amsterdam Public Health, Amsterdam UMC, Vrije Universiteit, Amsterdam, The Netherlands; c School of Psychological Sciences, College of Engineering, Science and Environment, University of Newcastle, Callaghan, Australia

**Keywords:** autonomic nervous system, HPA axis, inflammation, lifestyle, stress response

## Abstract

An unhealthy lifestyle has a critical role in the pathogenesis and course of several chronic disorders. It has been hypothesized that lifestyle may also impact biological systems involved in stress response. A global index of unhealthy lifestyle was calculated based on the cumulative presence of five self-reported lifestyle habits (smoking, excessive alcohol use, drug use, low physical activity and short sleep) in 2783 participants (18–65 years) from the Netherlands Study of Depression and Anxiety. The functioning of biological stress systems was based on multiple physiological measures of cortisol, inflammatory cytokines and autonomic cardiac activity. The unhealthy lifestyle index was associated with hyperactivity of hypothalamus–pituitary–adrenal axis and increased inflammation, indicating that with increasing unhealthy habits, the level of biological stress increases. No association with the autonomic nervous system activity was observed; however, the use of drugs increased parasympathetic cardiac activity and significantly impacted on ANS. Results were not impacted by a recent episode of depression or anxiety disorder. An unhealthy lifestyle may unfavorably impact on biological systems involved in stress response, which may underlie progression of several psychiatric as well as somatic chronic disorders.

## Introduction

In medicine, lifestyle refers to the set of habits and customs, including the use of substances such as alcohol and tobacco, dietary habits and exercise, which have important implications for health and are influenced by both environmental and heritable factors (Heller *et al.*, 1988).

Lifestyle is critically involved in the cause and maintenance of several noncommunicable and chronic diseases such as cancer, cardiovascular disease, diabetes, as well as neurodegenerative and psychiatric conditions (Sarris *et al.*, 2014; Arena *et al.*, 2017). Lifestyle is also responsible for the high comorbidity between mental and physical disorders (Möller-Leimkühler, 2010).

There is indeed a close relationship between mental disorders and lifestyle: those who suffer from them have a more unhealthy lifestyle, and interventions aimed at changing lifestyle can have therapeutic effects (Sarris et al., 2014, 2020; Walsh, 2011). Although the underlying mechanism is not uniformly clear, an unhealthy lifestyle seems to be associated with alterations of the biological systems of stress response (Lopresti *et al.*, 2013; Badini *et al*., 2020), alterations that are also present in several somatic diseases (McEwen, 1998).

The physiological response to stress involves a complex physiological system, located both in the central nervous system (hypothalamus and brainstem) and in the periphery of the body [outflow of the hypothalamic–pituitary–adrenal (HPA) axis and autonomous nervous system (ANS)]. Further, the HPA axis and ANS closely interact with the immune system, which is the third main actor of the stress response system (Nater *et al.*, 2013).

The functioning of these interacting systems, as mentioned above, is also influenced by lifestyle factors. As for the HPA axis, nicotine exposure provokes acute activation of the HPA system (Kishioka *et al.*, 2014). Alcohol also has activating effects and interacts with glucocorticoid receptors (Blaine and Sinha, 2017). Almost all abuses of drugs, including opioid and psychostimulant drugs, sedative hypnotics and cannabinoids, deeply impact on HPA-functioning, both in acute- and in long-term use (Sarnyai *et al.*, 2001). However, the regular use of nicotine, alcohol and drugs can lead to HPA-axis tolerance, resulting in blunted cortisol response to substances or stress (Sarnyai *et al.*, 2001; Blaine and Sinha, 2017). Acute exercise also increases cortisol levels, whereas regular physical activity, although upregulating the basal activity of the HPA axis, can decrease its reactivity in front of eliciting stimuli (e.g. stress), leading to an overall hyporeactivity of the HPA axis (Chen *et al.*, 2017). The relationship between sleep and HPA axis is less clear; however, several studies have shown higher levels of cortisol, especially in the evening and at awakening, in people who suffer from insomnia (Vgontzas *et al.*, 2001), in subjects exposed to experimental sleep restriction (Spiegel *et al.*, 1999) and those who sleep little habitually (Späth-Schwalbe *et al.*, 1993).

Likewise, the ANS is influenced by the use of nicotine, alcohol and drugs use, physical activity and sleep. Nicotine produces an increase of sympathetic activity: for instance, healthy smokers, although in a normal range, have a higher heart rate (HR) and blood pressure than healthy nonsmokers (Zhou *et al.*, 2016). Nevertheless, the sympathetic activity can be attenuated in chronic smokers (Middlekauff *et al.*, 2014). An excessive use of alcohol is associated with several cardiac disorders and this association is likely mediated by an increase of the sympathetic activity of the ANS, as seen in alcohol abuse (Gardner and Mouton, 2015) although not consistently (Boschloo *et al.*, 2011). Insomnia is also highly comorbid with cardiovascular disorder, potentially because poor sleep increases sympathetic ANS activity (Javaheri and Redline, 2017). On the contrary, regular physical activity (moderate-to-high) can decrease the sympathetic ANS activity in the long term (Lanfranchi and Somers, 2002).

Finally, as for the inflammatory system, smoking increases the levels of several circulating inflammatory markers and cytokines, such as tumor necrosis factor-alpha (TNF-α) and interleukin-1 (IL-1), -6 (IL-6) and -8 (IL-8), while reducing the levels of anti-inflammatory cytokines such as IL-10 (Rom *et al.*, 2013). Similarly, several substances of abuse interact with the immune system and alter neuroimmune gene expression and signaling (Cui *et al.*, 2014). Physical exercise also increases the release of inflammatory cytokines. However, the inflammatory response induced by regular physical exercise seems to be tissue-specific: increased in the skeletal muscles cells and reduced in the periphery and adipose tissue. Regular exercise, globally, can be associated with anti-inflammatory effects, mediated mainly by a decrease in the production and release of TNF-α in the periphery of the body (Peake *et al.*, 2015). Poor sleep is associated with an increase of inflammatory processes. In particular, sleep seems to be involved in TNF-α and IL-6 release (Grandner *et al.*, 2016). However, we previously reported an opposite association between long sleep duration and increased levels of IL-6 and C-reactive protein (CRP) (Prather *et al.*, 2015).

In previous investigations from the Netherlands Study of Depression and Anxiety (NESDA), we found evidence of underlying associations between lifestyle factors and dysregulations in these biological systems related to stress response, though not always consistently with previous literature or in the expected direction (Vreeburg *et al.*, 2009; Vogelzangs *et al.*, 2012; van Reedt Dortland *et al.*, 2013; Hu *et al.*, 2017; Phillips and Fahimi, 2018; Kuzminskaite *et al.*, 2020; Elnazer *et al.*, 2021).

However, the relevance of individual behaviors on biological systems implicated in several disorders is limited by the fact that lifestyle includes a number of habits, harmful and protective, which may have, as a whole, differential influence on these systems. Further, it is known that unhealthy lifestyle factors tend to correlate, and the presence of a risky habit predicts the occurrence of one or more other risky habits (Schuit *et al.*, 2002; Coups *et al.*, 2004; Poortinga, 2007; Silva *et al.*, 2013). The impact of multiple unfavorable lifestyle factors on mechanisms underlying disease risk can, therefore, be more informative than that of single behaviors (King *et al.*, 2015). Therefore, we assume that a lifestyle that is overall more unhealthy, understood as containing multiple unhealthy habits, can result in more substantial dysregulations in biological systems of stress response. Using data from the large-scale NESDA, we tested whether a summary lifestyle index, examining drug and excessive alcohol use, smoking, poor sleep and physical inactivity, is indeed linearly associated with dysregulations of the HPA axis, inflammation and autonomic nervous systems.

## Methods

### Study sample

Data were from the NESDA, a large ongoing longitudinal cohort study on 2981 adults (18–65 years). Study details were previously described (Penninx *et al.*, 2008). Briefly, respondents were recruited between September 2004 and February 2007 from the community, primary care and in specialized mental health care settings, including persons with a lifetime diagnosis of a depressive or anxiety disorder and healthy controls. Exclusion criteria were represented by: (a) a primary clinically overt diagnosis of other psychiatric conditions such as psychotic, obsessive compulsive, bipolar or severe addiction disorder, and (b) not being fluent in Dutch. Baseline data collection consisted of a medical examination, blood draw, self-report questionnaires and a detailed interview. Assessments were conducted by specially trained research staff. The research protocol was approved by the ethical committee of participating universities and all respondents provided written informed consent.

Of all participants, 2783 persons with self-reported lifestyle factors were included (93.4% of overall sample). For specific physiological stress markers, however, we had to exclude subjects with missing data for that assessment: 881 missing data for HPA-axis measures, 114 missing data for ANS and 31 missing data points for inflammatory markers.

### Measurements

#### Lifestyle factors

In this study, the following self-reported lifestyle factors were considered: smoking, alcohol use, use of drugs, physical activity and amount of sleep.

According to the original NESDA protocol (Penninx, *et al.*, 2008), participants self-assessed as current smoker, exsmoker and never smoker. The level of alcohol consumption was evaluated by the Alcohol Use Disorders Identification Test (Saunders *et al*., 1993) and classified as abstinence (0 drinks per week), moderate use (1–21 for males and 1–14 for females) or heavy use of alcohol (>21 for males and >14 for females), according to the Dutch alcohol use guidelines (Health Council of the Netherlands, 2015). The level of general physical activity was assessed by the International Physical Activity questionnaire (Ainsworth, *et al*., 2000; Craig, *et al*., 2003), which calculates an overall level of physical activity, weighted by the intensity of the activities. For drug use, people indicated whether they had used cannabis, ecstasy, speed, cocaine, heroin or LSD in the previous month. Finally, the amount of sleep was calculated as the average number of self-reported hours of sleep per night in the last 4 weeks.

Unhealthy behaviors were dichotomized as follows: current smoker, heavy use of alcohol, use of drugs in the past month, low level of physical activity and an average of less than 7 h of sleep. An index of unhealthy lifestyle was derived from the sum of each of the unhealthy behaviors (Hiles *et al.*, 2017). For descriptive purposes, subjects were grouped into three categories of lifestyle, according to the tertiles of the distribution of the unhealthy lifestyle index in the sample: healthy pattern (no self-reported unhealthy behavior), intermediate pattern (one self-reported unhealthy behavior) and unhealthy pattern (two or more self-reported unhealthy behaviors).

#### Biological stress measures

Methods of evaluation of HPA-axis function, inflammation levels and ANS were previously reported [see e.g. (Barakat *et al.*, 2012; Duivis *et al.*, 2013; Lamers *et al.*, 2013)].

Briefly, for HPA-axis functioning, respondents were instructed to collect seven saliva samples at home on a regular (preferably working) day using Salivettes (Sarstedt, Nümbrecht, Germany) at different time points. Cortisol analysis was performed by competitive electrochemiluminescence immunoassay (Roche, Basel, Switzerland) (van Aken *et al.*, 2003). We measured the cortisol awakening curve (cortisol curve during first hour after awakening) by calculating the area under the curve with respect to the ground (AUCg, *N* = 1737) and with respect to the increase (AUCi, *n* = 1077), the mean evening cortisol level (MEC, average of levels at 10 and 11 p.m., *N* = 1892) and the cortisol suppression ratio (CSR) after dexamethasone intake (by the ratio of cortisol values at awakening on the day before and the day after ingestion of 0.5 mg dexamethasone, *N* = 1789) [see (Vreeburg *et al.*, 2013) for more details]. The AUCg is an estimate of the total cortisol secretion over the first hour after awakening, whereas the AUCi is a measure of the dynamic of the cortisol awakening response over time, more indicative of the sensitivity of the system (Pruessner *et al.*, 2003); MEC levels are indicative of basal HPA-axis activity, because cortisol levels are generally low at the end of the day; the dexamethasone CSR examines the adequacy of the negative feedback of the HPA axis. A higher CSR indicates suppression by dexamethasone, which occurs when the feedback loop functions adequately, a lower ratio indicates nonsuppression of the HPA axis (Carroll, 1984).

For inflammation, circulating plasma levels of CRP (*N* = 2742), IL-6 (*N* = 2743) and TNF-α (*N* = 2726) were assessed from fasting blood samples [see (Vogelzangs *et al.*, 2012) for more details].

For ANS activity, the HR (*N* = 2669), the pre-ejection period (PEP, *N* = 2645) and respiratory sinus arrhythmia (RSA, *N* = 2669) were extracted from the combined dZ and ECG signals (Licht *et al.*, 2012). HR is an indicator of combined sympathetic and parasympathetic nervous system activity; PEP is a measure of cardiac sympathetic control (long PEP reflecting low cardiac sympathetic control) (Berntson *et al.*, 1994); RSA reflects cardiac parasympathetic (vagal) control (high RSA reflecting high cardiac vagal control) (Yasuma and Hayano, 2004; Karagueuzian, 2008). Cardiac autonomic balance was also calculated [RSA − PEP(*−1)] (high scores indicate parallel high parasympathetic and low sympathetic activity).

It is expected that the various biological stress indicators within the same system (HPA axis, inflammation and ANS) will be closely correlated; this expectation was confirmed in our sample (see also Table 1). Therefore, in order to globally evaluate the HPA-axis functioning, the inflammatory status and ANS activity, the scores of the markers in each system were standardized and averaged. Three global scores were, therefore, obtained: inflammation (mean of standardized scores of CRP, IL-6 and TNF-α), HPA axis (mean of standardized scores of AUCg, AUCi, MEC and CSR) and ANS (mean of standardized scores of HR, PEP and RSA). Since low CSR indicates less efficient negative feedback of the HPA axis, high PEP reflects low sympathetic activity and, at the opposite, high RSA indicates high parasympathetic activity, the standardized scores were reversed (*−1) before calculating the mean with the other parameters in each system.

**Table 1:** Correlations among biological measures of stress

Measures of stress		HPA axis				Inflammation			ANS			HPA-axis score^a^	Inflammation score^b^	ANS score^c^	CAB^d^
		AUCg	AUCi	MEC	CSR	CPR	IL-6	TNF-(	HR	RSA	PEP				
HPA axis	AUCg	–	0.48*	0.37*	0.21*	−0.02	−0.03	0.03	0.01	−0.09*	−0.02	0.64*	−0.03	0.06	−0.07
	AUCi	0.48*	–	0.07	−0.09*	−0.04	0.06	<0.01	0.01	−0.05	−0.04	0.61*	0.01	0.05	−0.05
	MEC	0.37*	0.07	–	−0.23*	−0.02	<0.01	0.01	<0.01	−0.10*	0.02	0.75*	0.02	0.04	−0.04
	CSR	0.21*	−0.09*	−0.23*	–	0.02	−0.04	−0.02	0.04	0.03	−0.04	−0.42*	−0.04	0.03	≤0.01
Inflammation	CPR	−0.02	−0.04	−0.02	0.02	–	0.34*	0.13*	0.20*	−0.15*	−0.12*	<0.01	0.71*	0.23*	−0.18*
	IL-6	−0.03	0.06	<0.01	−0.04	0.34*	–	0.15*	0.12*	−0.19*	−0.06*	≤0.01	0.67*	0.16*	−0.15*
	TNF-(	0.03	0.01	0.01	−0.02	0.13*	0.15*	–	0.01	−0.07*	<0.01	<0.01	0.48*	0.03	−0.02
ANS	HR	0.01	<0.01	<0.01	0.20*	0.20*	0.12*	0.01	–	−0.33*	−0.25*	0.01	0.17*	0.72*	−0.38*
	RSA	−0.09*	−0.10*	−0.10*	−0.15*	−0.15*	−0.19*	−0.07*	−0.33*	–	0.14*	−0.11*	−0.22*	−0.65*	0.76*
	PEP	−0.02	0.02	0.02	−0.12*	−0.12*	−0.06*	<0.01	−0.25*	0.14*	–	−0.01	−0.10*	−0.58*	0.77*

ANS, autonomous nervous system; AUCg, area under curve with respect to the ground; AUCi, area under the curve with respect to the increase; CAB, cardiac autonomic balance; CRP, C-reactive protein; CSR, cortisol suppression ratio (dexamethasone); HPA axis, hypothalamic–pituitary–adrenal axis; HR, heart rate; IL-6, interleukin-6; MEC, mean evening cortisol; PEP, pre-ejection period; RSA, respiratory sinus arrhythmia; TNF-α, tumor necrosis α.

a Mean of AUCg, AUCi, MEC and CSR^(*−1)^ standardized scores.

b Mean of CPR, IL-6 and TNF-α standardized scores.

c Mean of HR, RSA^(*−1)^ and PEP^(*−1)^ standardized scores.

d Formula: [RSA – PEP^(*−1)^] (high scores indicates parallel high parasympathetic and low sympathetic activity).

* Significant correlations at *P* < 0.001 (two-tailed).

### Covariates

Socio-demographic factors included sex, age and years of attained education. As a health indicator, we considered the number of self-reported chronic diseases (including cardiovascular diseases, diabetes, lung disease, osteoarthritis, rheumatic disease, cancer, ulcer, intestinal problem, liver disease, epilepsy, and thyroid gland disease). Further, according to previous studies, apart from standard covariates, other additional factors were taken into account. For analyses on inflammation variables, we assessed the use of systemic anti-inflammatory medication (M01A, M01B, A07EB and A07EC) (Vogelzangs *et al.*, 2012). For the HPA-axis analyses, awakening time, working status on the sampling day and season (categorized into dark months – October to February – and months with more daylight –March to September–) were considered (Vreeburg *et al.*, 2009). For ANS analyses, additional adjustments were made for respiratory rate (for RSA), and for mean arterial pressure to account for potential between-subject differences in afterload in PEP analyses (Houtveen *et al.*, 2005). Since participants with depression or anxiety disorder were oversampled in our cohort, and a recent episode of illness can influence biological stress measures, we evaluated recent episodes (in the last 6 months) of depressive (major depression, dysthymia) or anxiety disorder (panic disorder, agoraphobia, generalized anxiety disorder, and social phobia), as ascertained using the Composite Interview Diagnostic Instrument (CIDI version 2.1) (Andrews and Peters, 1998) (see section Statistical analyses for more details), as potential confounders of the association between biological stress measures and unhealthy lifestyle.

### Statistical analyses

Demographic and health-related characteristics were compared across each dichotomous lifestyle behavior as well as the unhealthy lifestyle patterns (healthy, intermediate and unhealthy, on the basis, respectively, of the absence, the presence of 1 or at least 2 self-reported unhealthy habits) using the Chi-square test, the Student’s *t*-test and the one-way analysis of variance.

Biological markers scores were not normally distributed. Therefore, natural logarithm-transformations were used in the analyses, and these values were presented back-transformed in the tables.

The associations between the lifestyle patterns (and dichotomous lifestyle behaviors) and each of the inflammatory, HPA axis, and ANS markers were tested in separate linear regression analyses. All analyses were adjusted for standard and additional covariates as described in the previous section.

The associations between the unhealthy continuous lifestyle index (and each dichotomous lifestyle factor) with HPA axis, ANS and inflammation global scores were tested in separate linear regression analyses adjusted for covariates. The effect of a recent episode of depression or anxiety as a moderating variable was quantified by regression analysis that is by regressing it, along with lifestyle index and their interaction with the outcome variables (HPA axis, ANS and inflammation index scores).

All statistical analyses were performed using SPSS v.23.0 (Statistics IS, 2015, Armonk, New York). A Bonferroni correction based on the number of the main dependent (three sets of correlated biological markers) and independent variables (two sets of correlated lifestyle factors) was applied (3 × 2 = 6 tests) and an alpha value of 0.008 was used to determine statistical significance. With these parameters, we had a sufficient power of more than 0.90 to detect small effect sizes (f^2^ < 0.01) in linear regression models with 5–10 predictors.

## Results

### Sample characteristics and lifestyle behaviors

The sample consisted of a total of 2783 participants; they were 947 (34%) males and 1836 (66%) females, aged 41.8 (±13.1) years.

The rates of unhealthy lifestyle factors in the whole sample were the following: 38.2% for smoking (*n* = 1064), 20.3% for excessive alcohol use (*n* = 566), 7.7% for drug use (*n* = 215), 23.2% for low physical activity (*n* = 645) and 24.8% for poor sleep (*n* = 690).

As expected, unhealthy factors tended to co-occur: smoking was significantly associated with more excessive alcohol use (Chi-sq = 98.88; *P* < 0.001), drugs use (Chi-sq = 139.36; *P* < 0.001) and low physical activity (Chi-sq = 23.47; *P* < 0.001). Excessive alcohol use was also associated with drug use (Chi-sq = 98.52; *P* < 0.001). Poor sleep was associated with low physical activity level (Chi-sq = 12.57; *P* < 0.001).

Participant characteristics, grouped into healthy (no unhealthy habits), intermediate (one unhealthy habit) and unhealthy lifestyle (two or more unhealthy habits) groups, for descriptive purposes only, are reported in Table 2. Subjects with a healthy and intermediate lifestyle were more likely to be females, more educated, with more favorable stress and inflammation profile and a lower number of chronic diseases than those with an unhealthy lifestyle. Those with a healthy lifestyle also made less use of anti-inflammatory medications. Finally, significantly lower rates of recent depressive and anxiety disorders were observed among subjects with a healthy lifestyle.

**Table 2:** Sample characteristics stratified for lifestyle categories

Sample characteristics		Lifestyle Categories (*n* = 2783)			*P*-value
		Healthy (*n* = 883)	Medium (*n* = 978)	Unhealthy (*n* = 922)	
Demographics					
Sex (female), *n* (%)		668 (36.4)	645 (35.1)	523 (28.5)	**<0.001**
Age (years), mean (SD)		41.1 (13.3)	41.7 (13.1)	42.4 (12.7)	0.14
Education level attained		12.9 (3.1)	12.1 (3.2)	11.7 (3.3)	**<0.001**
Health factors					
Number of chronic diseases, mean (SD)		0.8 (1.0)	0.9 (1.1)	1.0 (1.1)	**0.001**
Anti-inflammatory drugs (frequent use), *n* (%)		49 (19.4)	87 (34.4)	117 (46.2)	**<0.001**
Cardiac medication (yes), *n* (%)		112 (27.1)	159 (38.4)	143 (34.5)	0.08
Recent depressive/anxiety episode (positive), *n* (%)^a^		412 (25.6)	569 (35.4)	628 (39.0)	**<0.001**
Physiological stress systems^b,c^					
HPA-axis function, mean (SD)					
AUCg (nmol/l/h)	1737	16.71 (1.43)	17.55 (1.47)	19.05 (1.45)	**<0.001**
AUCi (nmol/l/h)	1077	3.03 (3.43)	3.72 (3.24)	4.06 (3.13)	0.01
Mean evening cortisol (nmol/l)	1892	4.01 (1.65)	4.63 (1.76)	5.55 (1.68)	**<0.001**
Cortisol suppression ratio	1789	2.59 (1.67)	2.38 (1.66)	2.26 (1.68)	**0.001**
Autonomic nervous system (ANS), mean (SD)					
Heart rate (bpm)	2669	71.63 (1.14)	71.12 (1.14)	71.35 (1.15)	0.59
Pre-ejection period (ms)	2645	117.57 (1.16)	118.54 (1.17)	119.93 (1.17)	**0.002**
Respiratory sinus arrhythmia (ms)	2669	39.79 (1.71)	37.97 (1.73)	37.18 (1.80)	0.19
Inflammation, mean (SD)					
C-reactive protein (mg/l)	2742	1.13 (3.49)	1.27 (3.32)	1.45 (3.54)	**0.001**
Interleukin-6 (pg/ml)	2743	0.80 (2.16)	0.84 (2.13)	0.90 (2.01)	0.02
Tumor necrosis factor-alpha (pg/ml)	2726	0.84 (1.90)	0.81 (1.90)	0.86 (1.84)	0.86

Bold fonts indicate significant associations.

AUCg, area under the curve with respect to the ground; AUCi, area under the curve with respect to the increase.

a Recent depression/anxiety diagnoses (past 6 months).

b Natural logarithm-transformed factors presented back-transformed.

c Controlled for covariates as explained in methods.

### Biological stress systems

Correlations among measures of the different biological stress systems are reported in Table 1. In the whole sample, strong correlations were observed for HPA-axis markers: AUCg, AUCi and MEC were all positively correlated, whereas CSR was negatively correlated with AUCi and MEC, though positively with AUCg. Inflammatory markers were all highly and positively correlated. Regarding the ANS, HR correlated negatively with RSA (high parasympathetic activity) and PEP (low sympathetic activity). RSA and PEP were positively associated, indicating an inverse correlation between sympathetic and the parasympathetic activation.

Further, correlations among markers within different biological systems were observed. Overall, inflammation markers were associated with high HR, high sympathetic (low PEP) and low parasympathetic activity (low RSA). A high basal and ‘output’ activity of the HPA axis (high MEC and high AUCg) were also correlated with a low parasympathetic activity (low RSA).

Correlations were similar in healthy subjects and those with a recent episode of major depression or anxiety disorder (data not shown).

### Lifestyle and biological stress systems

Associations between single lifestyle factors and each biological measure are shown in Table 3. All lifestyle factors were significantly associated at least with one biological marker, and in several cases with biological markers related to different systems. Use of all substances (smoking, alcohol and drugs) was associated with at least some HPA-axis measure; cigarette smoking and poor physical activity were associated with increased CRP and IL-6 levels. Finally, the ANS system was associated with all lifestyle factors, though nonsignificantly in some cases. Of interest, smoking was associated with increased PEP (lower sympathetic activity); drug use, sleep and, as a trend, excessive alcohol use, with increased RSA (higher parasympathetic activity); poor physical activity and, as a trend, poor sleep, with increased HR, whereas drugs use was associated with decreased HR.

**Table 3:** Associations between unhealthy lifestyle behaviors and single biological stress measures

Lifestyle factors	HPA axis				Inflammation			ANS		
	AUCg	AUCi	MEC	CSR	CRP	IL-6	TNF-α	HR	PEP	RSA
	B (95% CI), *P*-value	B (95% CI), *P*-value	B (95% CI), *P*-value	B (95% CI), *P*-value	B (95% CI), *P*-value	B (95% CI), *P*-value	B (95% CI), *P*-value	B (95% CI), *P*-value	B (95% CI), *P*-value	B (95% CI), *P*-value
Unhealthy lifestyle (index)	**0.06 (0.04–0.08**), **<0.001**	0.09 (0.02–0.16), 0.01^a^	**0.14 (0.11–0.16**), **<0.001**	−**0.05** (−**0.07 to** −**0.02**), **0.001**	**0.08 (0.03–0.12**), **0.001**	0.03 (<01–0.06), 0.02^a^	−0.01 (−0.03–0.02), .86	−0.01 (−0.01 to 0.01), 0.72	**0.01** (**0.01–0.02**), **0.006**	0.02 (−0.01 to 0.03), 0.06
Smoking	**0.17** (**0.13–0.21**) **<0.001**	**0.25** (**0.10–0.39**), **0.001**	**0.41** (**0.36–0.46**), **<0.001**	−**0.12** (−**0.17 to** −**0.06**), **<0.001**	**0.15** (**0.06–0.24**), **0.001**	**0.12** (**0.07–0.18**), **<0.001**	−0.01 (−0.05 to 0.04), 0.79	0.01 (−0.01 to 0.01), 0.75	**0.02** (**0.01–0.04**), **<0.001**	0.02 (−0.02 to 0.05), 0.35
Excessive Alcohol	**0.07** (**0.03–0.12**), **0.002**	0.12 (−0.06 to 0.30), 0.20	**0.10** (**0.03–0.16**), **0.004**	−0.02 (−0.08 to 0.05), 0.58	0.01 (−0.11 to 0.12), 0.90	0.01 (−0.08 to 0.06), 0.80	−0.01 (−0.07 to 0.05), 0.65	−0.01 (−0.02 to 0.01), 0.09	0.01 (−0.01 to 0.02), 0.30	0.04 (0.01–0.08), 0.04^a^
Drug use	0.05 (−0.02 to 0.13), 0.18	−0.11 (−0.39 to 0.16), 0.42	**0.22** (**0.12–0.33**), **<0.001**	−0.03 (−0.13 to 0.08), 0.64	−0.03 (−0.19 to 0.14), 0.76	−0.01 (−0.10 to 0.09), 0.86	−0.09 (−0.17 to 0.01), 0.06	−0.02 (−0.04 to −0.01), 0.02^a^	0.01 (−0.02 to 0.03), 0.56	**0.10** (**0.04–0.17**), **0.001**
Low physical activity	0.02 (−0.03 to 0.06), 0.64	−0.05 (−0.23 to 0.13), 0.56	0.05 (−0.01 to 0.11), 0.11	−0.03 (−0.09 to 0.03), 0.37	**0.29** (**0.18–0.40**), **<0.001**	0.05 (−0.01 to 0.12), 0.11	0.04 (−0.02 to 0.10), 0.17	**0.02** (**0.01–0.03**), **0.001**	−0.01 (−0.02 to 0.01), 0.79	−0.04 (−0.08 to 0.01), 0.07
Poor sleep	−0.03 (−0.06 to 0.01), 0.16	−0.06 (−0.20 to 0.09), 0.43	0.01 (−0.05 to 0.05), 0.98	−0.03 (−0.08 to 0.02), 0.28	0.07 (−0.01 to 0.16), 0.10	0.02 (−0.04 to 0.07), 0.56	0.02 (−0.02 to 0.07),.34	0.01 (0.01–0.02), 0.01^a^	0.01 (−0.01 to 0.02), 0.36	−**0.05** (−**0.08 to** −**0.02**), **0.004**

Bold fonts indicate significant associations.

ANS, autonomous nervous system; AUCg, area under curve with respect to the ground; AUCi, area under the curve with respect to the increase; CRP, C-reactive protein; CSR, cortisol suppression ratio (dexamethasone); HPA axis, hypothalamic–pituitary–adrenal axis; HR, heart rate; IL-6, interleukin-6; MEC, mean evening cortisol; PEP, Pre-EJECTION period; RSA, respiratory sinus arrhythmia; TNF-α, tumor necrosis α.

a Trends of association.

**Fig. 1:**
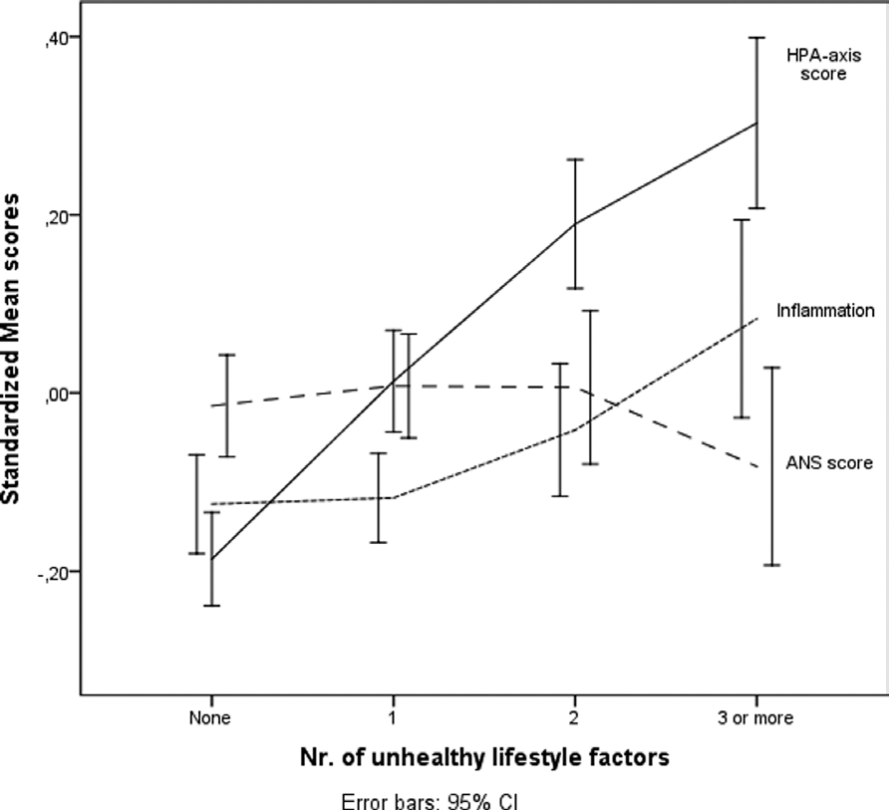
Mean standardized biological systems scores stratified for the number of unhealthy lifestyle factors. HPA axis, hypothalamic–pituitary–adrenal axis; ANS, autonomous nervous system.

When considering the cumulative biological stress scores, the unhealthy lifestyle index (continuous total number of unhealthy behaviors) was positively associated with the HPA axis and the inflammation systems, but not with the ANS factor (Table 4). As shown in Fig. 1, the HPA-axis (hyperactivity) index and the inflammation index increased with the increase in the number of unhealthy lifestyle factors.

**Table 4:** Association between unhealthy lifestyle and lifestyle factors with biological systems involved in stress response

Lifestyle factors	HPA-axis score^a^	ANS score^b^	Inflammation score^c^
	B (95% CI), *P*-value	B (95% CI), *P*-value	B (95% CI), *P*-value
Unhealthy lifestyle (index)	**0.16** (**0.12–0.19**), **<0.001**	−0.003 (−0.05 to <0.01), 0.09	**0.04** (**<0.01–0.06**), **0.008**
Tobacco smoking	**0.45** (**0.38–0.51**), **<0.001**	−0.03 (−0.09 to 0.02), 0.26	**0.09** (**0.04–0.15**), **<0.001**
Excessive alcohol intake	**0.14** (**0.05–0.22**), **0.002**	−0.04 (−0.11 to 0.03), 0.24	<0.01 (−0.06 to 0.07), 0.90
Drug use	**0.20** (**0.06–0.33**), **0.007**	−**0.14** (−**0.25 to 0.04**), **0.009**^d^	−0.03 (−0.13 to 0.06), 0.51
Low physical activity	0.04 (−0.04 to 0.12), 0.31	0.08 (0.01−0.15), 0.02 ^d^	**0.11** (**0.05–0.17**), **<0.001**
Poor sleep	−0.02 (−0.09 to 0.04), 0.47	0.05 (<0.01–0.11), 0.08	0.04 (<−0.01 to 0.09), 0.10

Bold fonts indicate significant associations.

ANS, autonomous nervous system; AUCg, area under curve with respect to the ground; AUCi, area under the curve with respect to the increase; CRP, C-reactive protein; CSR, cortisol suppression ratio (dexamethasone); HPA axis, hypothalamic–pituitary–adrenal axis; HR, heart rate; IL-6, interleukin-6; MEC, mean evening cortisol; PEP, pre-ejection period; RSA, respiratory sinus arrhythmia; TNF-α, tumor necrosis α.

a Mean of AUCg, AUCi, MEC and CSR(*−1) standardized scores.

b Mean of HR, RSA(*−1) and PEP(*−1) standardized scores.

c Mean of CPR, IL-6 and TNF-α standardized scores.

d Trends of association.

### Moderation analysis

Post hoc regression models were performed in order to control the potential moderator effect of a recent episode of depression or anxiety disorder on identified associations. According to these analyses, a recent episode (1609 subjects, 57.8% of the sample, which was associated with lifestyle) did not influence the association between the unhealthy lifestyle index and the HPA axis (unhealthy lifestyle × recent episode: *P* = 0.59), the ANS (*P* = 0.93) and the inflammation system (*P* = 0.72).

## Discussion

According to previous large evidence of an impact of individual lifestyle factors (smoking, alcohol, etc.) on the biological systems involved in stress response, we hypothesized that a growing involvement in multiple risky habits would be associated with increasing dysregulations at the level of these systems. Our data support this hypothesis. Indeed, an increasing unhealthy lifestyle was associated with more HPA-axis dysregulations and higher systemic inflammation. Further, the use of drugs of abuse significantly impacted autonomic cardiac activity, but the association with a number of unfavorable lifestyle indicators did not show a linear trend with ANS dysregulation.

HPA-axis dysregulations were mainly associated with tobacco smoking and, to a lower extent, with excessive alcohol intake and recent drug use. These findings are consistent with the known effect of nicotine, alcohol and virtually all substances of abuse, on glucocorticoid signaling, leading to stress-related neuroadaptations of the HPA axis (Rohleder and Kirschbaum, 2006; Edwards *et al.*, 2015; Fosnocht and Briand, 2016). Substance-induced stress reactivity is probably a mechanism contributing to addictive phenotypes (Lovallo, 2006; Fosnocht and Briand, 2016).

Consistently with previous analyses on this same sample of individuals (Hu *et al.*, 2017), smoking and drugs were associated with a predominant parasympathetic activation. Cigarette smoking, as well as acute administration of common drugs that activate brain reward pathways, are expected to activate the ANS. However, regular and chronic uses of these drugs are associated with autonomic adaptations leading to reduced sympathetic response and blunted autonomic responses. These adaptations are thought to be involved in the maintenance of substance use (Sinha, 2008).

In line with a recent analysis on this same sample (Hu *et al.*, 2017), poor physical activity increased HR, confirming the negative effects of sedentary behaviors (vs. active life) on autonomic cardiac regulation (Besnier *et al.*, 2017). Moreover, we also found nonsignificant trends of autonomic activation associated with poor sleep (high HR and low RSA), in agreement with findings supporting sympathetic hyperarousal in insomnia (Roth *et al.*, 2007).

The unhealthy lifestyle, especially tobacco smoking and a low level of physical activity, were significantly associated with higher inflammation levels. Accordingly, smoking and physical inactivity, along with a range of other lifestyle factors such as psychosocial stress, poor diet and sleep, have been associated with systemic inflammation, especially the one related to depressive conditions (Berk *et al.*, 2013).

Anxiety disorders and depression are strongly characterized by alterations of biological systems of stress response (Maes *et al.*, 1998; Raison *et al.*, 2006; Sgoifo *et al.*, 2015). Our sample was composed of more than half of patients with a recent or current depressive or anxious episode. However, recent episodes did not moderate the associations between unhealthy lifestyle and biological dysregulations, further supporting the significance of lifestyle factors in disorders characterized by dysregulation of stress response systems.

Medications may have a complex effect on lifestyle and inflammation, antidepressants may decrease inflammation and increase activity in depression but also potentially induce weight gain; therefore, a concomitant psychoeducation treatment is strongly suggested (El-Khoury *et al.*, 2020; Hidalgo-Mazzei *et al.*, 2020; Benedetti *et al.*, 2022; Dörks *et al.*, 2022).

## Limitations and strengths of the study

Among limitations, this cross-sectional study was unable to capture the high variability and reciprocal interactions of physiological stress markers, and to suggest possible causal paths linking lifestyle and biological dysregulations. The compliance with saliva sampling might have been inaccurate, and although hardly feasible in large cohorts, multiple sampling days would have enhanced the HPA function assays. Self-report of lifestyle is also prone to inaccuracy and bias. Unfortunately, no data about dietary patterns, a relevant lifestyle factor [e.g. (Quirk *et al.*, 2013)], was collected at the time of biological evaluation.

Strengths of the present study are represented by its large sample size, the collection of a range of unhealthy lifestyle factors, enabling us to derive a global unhealthy lifestyle index, and the collection of relevant socio-demographic, health-related and psychiatric variables in a large adult age range. This is, to our knowledge, the first study to link a range of unhealthy lifestyle factors with the major physiological stress systems and to shed light on the cumulative effect of multiple lifestyle factors on stress markers.

## Conclusion

Present findings confirm an association between the unhealthy lifestyle and dysregulations of the biological systems involved in stress response. The biological effects mediated by lifestyle may account for a significant proportion of the environmental risk for several noncommunicable and chronic disorders, and, therefore, be susceptible to preventive as well as therapeutic interventions.

## Acknowledgements

### Conflicts of interest

There are no conflicts of interest.
